# Conserved RNA Binding Activity of Phosphatidyl Inositol 5-Phosphate 4-Kinase (PIP4K2A)

**DOI:** 10.3389/fmolb.2021.631281

**Published:** 2021-05-28

**Authors:** Jatin Behari, Pranita Borkar, Arya Vindu, Vishal Dandewad, Sindhuri Upadrasta, Dhanasekaran Shanmugam, Vasudevan Seshadri

**Affiliations:** ^1^ National Centre for Cell Science, Pune, India; ^2^ Department of Biotechnology, SPPU, Pune, India; ^3^ CSIR-National Chemical Laboratory, Pune, India; ^4^ Academy of Scientific and Innovative Research, Ghaziabad, India

**Keywords:** RNA–protein interaction, translation regulation, malaria, posttranscriptional gene regulation, PIP4K2A

## Abstract

*Plasmodium falciparum* is a causative agent for malaria and has a complex life cycle in human and mosquito hosts. During its life cycle, the malarial parasite *Plasmodium* goes through different asexual and sexual stages, in humans and mosquitoes. Expression of stage-specific proteins is important for successful completion of its life cycle and requires tight gene regulation. In the case of *Plasmodium*, due to relative paucity of the transcription factors, it is postulated that posttranscriptional regulation plays an important role in stage-specific gene expression. Translation repression of specific set of mRNA has been reported in gametocyte stages of the parasite. A conserved element present in the 3′UTR of some of these transcripts was identified. Phosphatidylinositol 5-phosphate 4-kinase (PIP4K2A) was identified as the protein that associates with these RNA. We now show that the RNA binding activity of PIP4K2A is independent of its kinase activity. We also observe that PIP4K2A is imported into the parasite from the host on *Plasmodium berghei* and *Toxoplasma gondii.* The RNA binding activity of PIP4K2A seems to be conserved across species from *Drosophila* and *C. elegans* to humans, suggesting that the RNA binding activity of PIP4K may be important, and there may be host transcripts that may be regulated by PIP4K2A. These results identify a novel RNA binding role for PIP4K2A that may not only play a role in *Plasmodium* propagation but may also function in regulating gene expression in multicellular organisms.

## Introduction

*Plasmodium falciparum* is one of the causative agents for human malaria. The parasite is transmitted to humans with an infected mosquito bite that injects *Plasmodium* in the form of sporozoites. These sporozoites then infect the liver cells and multiply within them in the liver stage of the infection. The infected liver cells then release merozoites, which can no longer infect the liver cells, but can infect the red blood cells (RBC). The *Plasmodium* multiplies inside the infected RBC, and some of the infected RBCs then develop male and female gametocytes which are taken by the mosquitoes when they bite an infected human. In the mosquito, the male and female gametes combine to begin a sexual diploid stage of the parasite. The parasite then undergoes several morphological changes and exists as a haploid sporozoite in the salivary glands of the mosquito to restart the human stage of infection. During this complex life cycle, the parasite undergoes several morphological changes and encounters different temperatures and environments, including varied host cells. In order to be successful, the parasite extensively reprograms its gene expression many times. It has been suggested that one of the ways in which this reprogramming is achieved in the parasite are through posttranscriptional gene regulation, including regulation of protein synthesis and degradation (Coulson et al., 2004).

Parasite-specific translation regulation has been previously suggested, and it was observed that many transcripts are synthesized in a particular stage of the parasite but are translated only in the subsequent stage of the parasite (Le Roch et al., 2004). A conserved element in some of the translationally regulated transcripts that were transcribed in the gametocyte stage but are translated in the ookinete stage was identified in *Plasmodium berghei* (Hall et al., 2005). DDX6, a *Plasmodium* RNA helicase, was identified to be one of the proteins that play an important role in this process (Mair et al., 2006). Additional components of this translation regulation complex were identified by co-immunoprecipitation, including CITH, PABP, and eIF4E (Mair et al., 2010). More recently, polysome profiling of parasite transcripts has identified a large number of transcripts that are translationally regulated at different stages of the parasite (Bunnik et al., 2013). The 3′UTR of one of the translationally regulated RNA was used as bait to identify human phosphatidylinositol 5-phosphate 4-kinase (PIP4K2A) as a protein that specifically associates with *Plasmodium falciparum* transcripts (Vindu et al., 2018).

PIP4Ks are the enzymes which phosphorylate phosphatidylinositol 5-phosphate (PI5P) at the 4th position, leading to the synthesis of phosphatidylinositol 4,5-bisphosphate (PI4,5P_2_) (Rameh et al., 1997). It has been suggested that the major role of PIP4K2 kinase is in regulating the levels of PI5P since the major proportion of PI4,5P_2_ is synthesized by phosphorylation of PI4P at 5th position by the enzyme phosphatidylinositol 4-phosphate 5-kinase (PIP5K). The gene encoding PIP5K is found in all sequenced eukaryotic genomes while the gene for PIP4K was absent in unicellular eukaryotes like *Plasmodium* and *S. cerevisiae*. While lower eukaryotes like *D. melanogaster* and *C. elegans* seem to have a single isoform of PIP4K, the metazoans including humans have three isoforms of PIP4K known as PIP4K2A, PIP4K2B, and PIP4K2C, also known as 2α, 2β, and 2γ, respectively. It has been suggested that the single copy of PIP4K evolved into multiple isoforms through gene duplication (Khadka and Gupta, 2019). PIP4K in *Drosophila* has been shown to be important for proper larval development. PIP4K mutant *Drosophila* showed impaired mToR signaling with reduced S6K phosphorylation, altered insulin signaling, and reduced larval weight (Gupta et al., 2013). Different isoforms of mammalian PIP4K have been shown to be localized to different intracellular regions and regulate distinct cellular processes. PIP4K2A has been shown to be predominantly cytosolic and regulate cell proliferation and tumor progression (Ciruela et al., 2000; Jude et al., 2015; Lima et al., 2015, 2019; Liao et al., 2016; Shin et al., 2019; Zhang et al., 2019). PIP4K2B is mainly nuclear and is also involved in regulating cell proliferation and tumor progression (Wang et al., 2010; Kouchi et al., 2011; Keune et al., 2013; Xu et al., 2016; Chauvin et al., 2018). PIP4K2C is localized predominantly to the endomembrane and has been shown to regulate autoimmune response and autophagy (Al-Ramahi et al., 2017). Although all three mammalian isoforms have significantly different catalytic activity *in vitro*, all of them are able to complement the *Drosophila* PIP4K mutant (Mathre et al., 2019).

The regulatory roles of PIP4K has been mainly attributed to its role as a kinase. However, recently, a kinase-independent role for PIP4K has been suggested. It was shown that the expression of the kinase dead mutant in *Drosophila* was able to rescue the increase in the levels of PIP3 that was seen in PIP4K mutants (Sharma et al., 2019). Similarly, the kinase-dead mutant was also able to rescue the early endosome expansion in PIP4K *Drosophila* mutants (Kamalesh et al., 2017). These results suggest that, apart from its kinase function, PIP4K could have additional activities that could play an important role in specific cellular processes. We have previously shown that human PIP4K2A has RNA binding activity and can associate with specific *Plasmodium* parasite transcripts (Vindu et al., 2018). We now characterize the RNA binding activity of PIP4K2A and show that the kinase domain dead mutant still retains the RNA binding activity. We further show that importing PIP4K2A from the host cells to the parasite is also seen in the case of *Plasmodium berghei* and *Toxoplasma gondii*, and the RNA binding activity of PIP4K is conserved across species.

## Materials and Methods

### *Plasmodium berghei* Culture

BALB/c mice of 6–8 weeks were infected with *P. berghei* ANKA MRA 311 strain. The parasites were diluted using acid citrate dextrose (ACD) (2% sodium citrate, 0.84% NaCl), an anticoagulant. The culture was diluted so that it gives 5*10^6^ parasite/ml. 0.2 ml of this suspension (1*10^6^) was injected in an animal using the Intra peritoneal route. The parasitemia was assessed on alternate days by preparing smear from the tail vein followed by staining with acridine orange (10 μg/ml). When parasitemia reached 20–30%, the blood was collected using retro-orbital bleeding in ACD.

### Saponin Lysis for Isolation of *Plasmodium berghei* Parasite From RBCs

Parasite-infected RBCs were harvested from mice with retro-orbital bleeding by centrifugation at 250 *g* for 5 min with low deceleration. The supernatant was discarded, and the pellet was washed with PBS once. Then the pellet was again resuspended in PBS in 1:10 ratio, and saponin was added to the final concentration of 0.05% to lyse the RBCs. It was kept at RT for 10 min, and the released parasite was then collected by centrifuging it at 1000 g for 10 min without brakes. The supernatant was discarded carefully without disturbing the pellet. The pellet was washed with PBS twice so as to remove RBC contaminants and residual saponin. These pellets were then either stored in -80°C till further use for lysate preparation or were smeared on to coverslips and fixed, and then stored at 4°C.

### Toxoplasma Culture

The Type I RhΔku80 strains were used and propagated as follows. Human foreskin fibroblast (HFF) cells were used as host cells and were grown in a Dulbecco’s modified eagle medium (DMEM-high glucose) supplemented with 10% heat-inactivated fetal bovine serum, 2 mM GlutaMAX, 25 mM HEPES, and 50 μg/ml Gentamycin. Initially, the HFF cells were passaged and maintained in an incubator at 37°C and 5% CO_2_. Once the HFF cells formed confluent monolayers, the culture medium was changed to the parasite growth medium which is similar to the host cell medium except that it lacks serum. Then roughly 10^5^ RhΔku80 parasites were allowed to infect the HFF cells and incubated for 48 h. After 48 h, the parasites were harvested by scrapping the host cell monolayer and then passing the suspension through a 22-gauge needle to disrupt the host cells completely. Later, the parasites were separated from the host cells by filtering the suspension through a syringe filter fitted with a 3-µM nucleopore membrane (Whatman, GE Healthcare, United States). This allows the separation of host cells from the parasites, which are obtained in the filtrate. The filtrate is centrifuged at 1,200 g for 5 min to allow for the formation of the parasite pellet which is used for further work.

For immunocytometry, 6-well plates containing coverslips were initially seeded with HFF cells and incubated at 37°C and 5% CO_2_ until confluent. 10^4^ parasites were infected per well of the plate. After 24 h, the coverslips were fixed and processed for microscopy.

### Preparation of Cell Extracts

The infected RBC lysate and saponin-lysed parasite pellet were resuspended into three volumes of RSB (10 mM Tris pH7.5, 2.5 mMMgCl2, 150 mM NaCl, 0.5% NP40, 0.5% Trition x 100,1 mMPMSF, 1 mMDTT, and 1X PI). It was passed through an insulin syringe 10 times and kept on ice for 15 min, followed by 15 min spinning at 12000 g in a microfuge. The supernatant was collected as a lysate. The protein was estimated by Bradford method, and lysates were subjected to Western blotting**.**

The HFF cells and parasite pellets were washed once with PBS and then resuspended into 100 uL of RIPA lysis buffer (Pierce RIPA Buffer, 89,901). The pellets were then subjected to pipetting for 20 counts and kept on ice throughout the lysis. Alternatively, the pellets were also subjected to intermittent vortexing and then kept on ice. This process takes place for 30 min. After 30 min, the protein lysate was centrifuged at high speed, that is, 14,000 rpm, for 45 min. Protein content was estimated in the lysate using the Bradford method and analyzed by Western blotting.

#### Immunofluorescence

Toxoplasma-infected HFFs on a coverslip were fixed by the addition of formaldehyde (final concentration 4%) and incubated for 60 min followed by three washes with PBS. It was followed by permeabilization with 0.25%/0.1% of Triton X100. Blocking was performed with 2% BSA for 1 h followed by incubation with the primary PIP4K2A antibody (Santacruz sc100406) or PBS (secondary ab control) O/N at 4°C. The coverslips were washed thrice with PBS followed by incubation with the labeled secondary antibody (goat anti-mouse Alexa Fluor 488 Invitrogen A11001) for 1 h followed by three washes with PBS. The coverslips were stained with DAPI for 10 min and washed with PBS 3 times, and mounted on the slides. The samples were imaged on a Zeiss Axio observer microscope 7 with apotome 2.0, and the imaging was done using a 63X oil immersion objective lens. The images were processed using Zen 2 (blue edition) software.

For *P. berghei* immunostaining, a similar protocol was followed, except that the host RBCs were lysed by saponin to release the parasite, and the purified *P. berghei* was smeared on a slide and fixed with PBS containing 4% PFA and 0.0075% glutaraldehyde for 15 min. The slides were washed thrice with PBS and were incubated with sodium borohydride for 15 min to neutralize the cross-linking agent, followed by PBS wash. Here, ab-154585 anti-PIP4K2A antibody was used.

#### Expression and Purification of Recombinant Proteins

Recombinant PIP4K2A-WT, PIP4K2AG131L Y138F, D-PIP4K, PPK-2, and zPIP4K were expressed using BL21-codon + competent cells. At (OD600∼0.6–0.8), the cultures were transferred to 18°C, and protein expression was induced using 0.5 mM IPTG for 16 h. Proteins were further purified using nickel chelating resin (G-Biosciences) or HisTrap FF columns (GE Healthcare) in a high salt buffer (500 mM NaCl, 10 mM Tris HCL (pH 7.5 at 4°C), and 10% glycerol). Purified proteins were pooled and further purified using size-exclusion chromatography (superdex 200, 16/600).

#### Competitive RNA-EMSA

50 fmoles of radioactively labeled or 5-pico moles of fluorescently labeled (cy5.5) 3′UTR RNA and 11 p moles or 50 p moles of purified recombinant protein, respectively, were preincubated with 5 µg yeast tRNA, 40 units of RNAse inhibitor, 10-fold excess of self-cold competitor (self comp.), and 10-fold excess of nonspecific RNA (non self comp.) in a 1X binding buffer (30 mM KCl, 1 mM MgCl2, 40 mM Tris pH 8, 0.01% NP40, and DTT 1 mM) for radioactive EMSA or (500 mM NaCl, 10 mM Tris pH 7.5, and 10% Glycerol) for fluorescently labeled EMSA for 60 min at 16°C. The RNA–protein complex was resolved on 4% Native PAGE at 100 V for 8 h or 1% agarose gel at 100 V for 140 min. The gel was imaged by autoradiography (Typhoon FLA 9500) or by detecting fluorescence using LICOR. Bound fraction of 3′UTR (RNA) was calculated by densitometric analysis using ImageJ software.

#### Estimating the Dissociation Constant (K_d_) of PIP4K2A and RNA

About 50 fmoles of radioactively labeled or 5 pmoles of fluorescently labeled (cy5.5) 3′UTR RNA and increasing concentration (271nM–2173.91 nM) of purified recombinant protein (PIP4K2A-WT and PIP4K2AG131L Y138F) were preincubated with 5 µg yeast tRNA and 40 units of RNase inhibitor in a 1X binding buffer (30mM KCl, 1mM MgCl2, 40 mM Tris pH 8, 0.01% NP40, and DTT 1 mM) for radioactive EMSA or (500 mM NaCl, 10 mM Tris pH 7.5, and 10% glycerol) for fluorescently labeled EMSA for 60 min at 16°C. The RNA–protein complex was resolved on 4% Native PAGE at 100 V for 8 h or 1% agarose gel at 100 V for 140 min. Bound fraction of 3′UTR (RNA) was calculated by densitometric analysis using Image J software, and the mean (±SEM; *n* = 3) was plotted against increasing concentration of PIP4K2A. For the calculation of K_d_, half the bound fraction of RNA was extrapolated on the *x*-axis (concentration of protein) (Heffler and Walters, 2012).

#### Competitive RNA-EMSA Using Increasing Amount of Unlabeled Self-Cold Competitor

5 pmoles of fluorescently labeled (cy5.5) 3′UTR RNA and 50 pmoles of purified recombinant protein (PIP4K2A-WT, PIP4K2AG131L Y138F), with increasing amounts of unlabeled RAD51 3′UTR or RAD51 short, were preincubated with 5 µg yeast tRNA and 40 units of RNase inhibitor in a 1X binding buffer (500 mM NaCl, 10 mM Tris pH 7.5, and 10% Glycerol) for 60 min at 16°C. The RNA–protein complex was resolved on 1% agarose gel at 100 V for 140 min. The bound fraction of 3′UTR (RNA) was calculated by densitometric analysis using Image J software, and the means (±SEM; *n* = 3) was plotted against the increasing concentration of the unlabeled competitor.

#### *In Vitro* Pull-Down Assay

PIP4K2A pET28a + plasmid DNA and eIF4EBP1 pGEX-6P-1 were co-transformed into BL21-codon + competent cells; a single colony was inoculated into 5 ml SOC broth and grown for 3 h (OD600∼0.6–0.8). The culture was transferred to 18°C, and protein expression was induced by the addition of 0.5 mM IPTG for 12–16 h. Protein expression was assessed by comparing the un-induced and induced protein lysate. GST eIF4EBP1 was affinity-purified using glutathione resin (G bioscience) and washed several times to remove the nonspecifically bound proteins. The presence of His PIP4K2A was detected either by anti-His (SC-8036) or anti-PIP4K2A antibody (SC-100406). The same procedure was used to detect interaction between His PIP4K2A and only GST protein as a control.

### PIP4K2A Co-Immunoprecipitation

HEK293T cells overexpressing PIP4K2A were resuspended in a low salt buffer (10 mM Tris pH 7.5, 150 mM NaCl, and 10% glycerol) and lysed using lysozyme and multiple freeze–thaw cycles. The lysate was further subjected to sonication (Sonics Vibro Cell) with 2 s on and 5 s off at 80% amplitude for 1 min, followed by centrifugation at 12,000 *g* for 15 min at 4°C. The supernatant was collected, and PIP4K2A IP was done using 10 µg of PIP4K2A antibody (SC-100406). The magnetic bead (Protein G Mag Sepharose Xtra (cat no. 10244934)) bound antibody was mixed with lysate and incubated at 4°C for 16 h on rotation. The beads were then magnetically separated to remove the flow-through and washed with buffer three times, and eluted in 1X SDS loading dye. Here, mouse IgG was used as control pull-down. Control and PIP4K2A pull-down were loaded on 15% SDS PAGE along with input and probed with anti-PIP4K2A (CST#5527) and anti-eIF4EBP1 (CST #9644) antibodies.

### Luciferase Assay

PIP4K2A, along with firefly luciferase containing either RAD51 3′UTR or control 3′UTR in pESCLeu vector, was transformed into a W303a yeast strain. The transformants were selected on the plates lacking leucine. Single colonies of transformed W303a were inoculated into 25 ml SD media (−Leu, +ampicillin) and grown overnight at 30°C. After reaching OD600∼0.6 to 1, the culture was pelleted and washed with SD media w/o glucose. The culture was resuspended in SD media w/o glucose to OD 1, followed by induction using 2% galactose and grown for 24 h at 30°C. After 24 h of induction, 1 ml of the culture was removed, pelleted, and washed with glass-distilled water. The cell pellet was resuspended into a 500 μL lysis buffer (GeneCopoeia), and an equal amount of glass beads (500 μm diameter) was added and lysed using a SoniBeast™ cell disruptor (as per the manufacture’s guidelines). The lysate was centrifuged at 12,000g for 10 min, and the supernatant was collected and was used for estimating the luciferase activity by the steady glow luciferase assay (GeneCopoeia) in a Glomax (Turner BioSystem).

### Statistical Analysis

All the experiments were repeated atleast three times, and a representative image is shown for each experiment. Data were expressed as the mean of atleast three repetitions of independent experiments. The error bars indicate the standard error of mean. Data comparison was performed using unpaired *t*-test, one tailed using GraphPad prism software. The stars (*) indicate the upper boundary of *p* value (**** = *p* < 0.0001, *** = *p* < 0.001, ** = *p* < 0.01, and ns (nonsignificant) = *p* > 0.1).

## Results

### Kinase Domain Mutants of PIP4K2A Retain Their RNA Binding Activity

**FIGURE 1:**
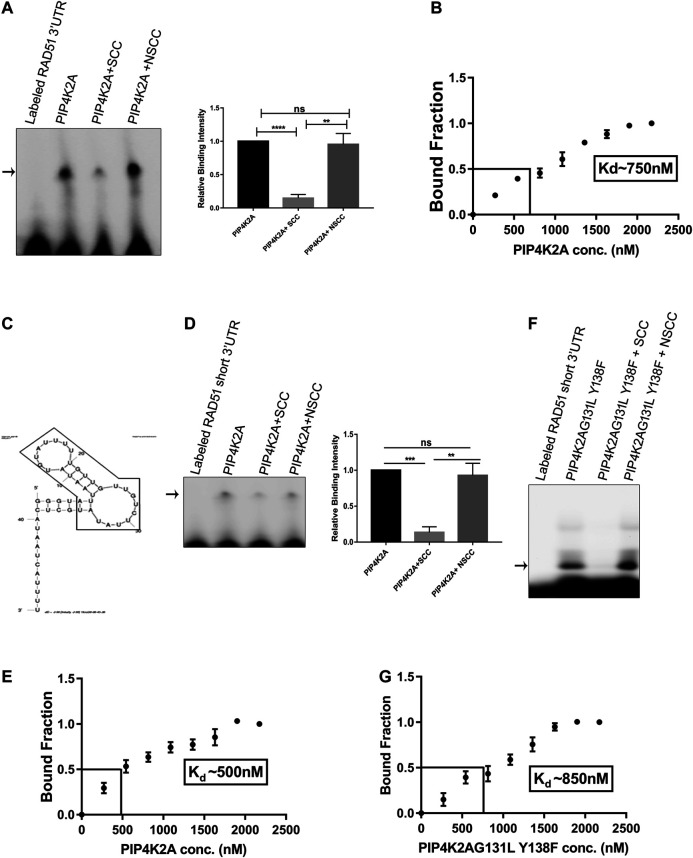
PIP4K2A binds specifically with RAD51 3′UTR, and RNA binding activity is independent of kinase activity. **(A)**. PIP4K2A and labeled 3′UTR of RAD51 were incubated at 16°C in 1x GSB for 1 h in the presence or absence of 10-fold excess of unlabeled self- or non-specific competitor. The reaction was then resolved on a 4% polyacrylamide gel for 8 h at 4°C, and the gels were scanned in a LI-COR scanner. The shifted bands were quantified using Image J software, and the mean (± SEM) relative band intensity was plotted (right panel), *n* = 3; unpaired *t*-test, one tailed, **** = *p* < 0.0001, ** = *p* < 0.01, and ns = *p* > 0.1. **(B).** RNA EMSA was performed with RAD51-3ÚTR and varying amounts of PIP4K2A, the band intensity was quantified using Image J software, and the mean (± SEM) ratio of bound to unbound fraction was plotted for each PIP4K2A concentration (*n* = 3). The value of K_d_ was obtained by extrapolating 50% bound fraction on the *X*-axis (PIP4K2A conc.) **(C)**. The predicted secondary structure of RAD51 3′UTR RNA indicating the formation of a stem loop structure by MFold algorithm. The RAD51-short sequence is boxed inside the arrow shape. **(D).** PIP4K2A and labeled RAD51-short were incubated at 16°C in 1x GSB for 1 h in the presence or absence of 10-fold excess of unlabeled self- or non-specific competitor. The reactions were then resolved on a 1% agarose gel at 4°C, and the gels were scanned in a LI-COR scanner. The shifted bands were quantified using ImageJ software, and the mean (± SEM) relative band intensity was plotted (right panel) (*n* = 3); unpaired *t*-test, one tailed, *** = *p* < 0.001, ** = *p* < 0.01, and ns = *p* > 0.1). **(E).** RNA EMSA was performed with RAD51-short RNA and varying amounts of PIP4K2A, the band intensity was quantified, and the mean (± SEM) ratio of bound to unbound fraction was plotted for each PIP4K2A concentration. The value of K_d_ was obtained by extrapolating 50% of bound fraction on the *X*-axis (*n* = 3). **(F).** PIP4K2A^G131L Y138F^, the kinase-dead mutant, and labeled RAD51-short were incubated at 16°C in 1x GSB for 1 h in the presence or absence of 10-fold excess of unlabeled self- or non-specific competitor. The reactions were then resolved on a 1% agarose gel at 4°C, and the gels were scanned in a LI-COR scanner. **(G)**. RNA EMSA was performed with RAD51-short RNA and varying amounts of PIP4K2A^G131L Y138F^, the band intensity was quantified, and the mean (±SEM) ratio of bound to unbound fraction was plotted for each PIP4K2A ^G131L Y138F^ concentration (*n* = 3). The value of K_d_ was obtained by extrapolating 50% bound fraction on the *X* axis.

Previously, we have shown that hPIP4K2A binds to *Plasmodium* transcripts and is important for efficient parasite propagation. We assessed the specificity of the interaction by competitive RNA EMSA assay. Incubation of the purified PIP4K2A with labeled 3′UTR of *Plasmodium* Rad51 transcripts resulted in the formation of a specific shifted band. The shifted band could be competed out in the presence of unlabeled 3′UTR of *Plasmodium* Rad51 but not by nonspecific control RNA (Figure 1A). We further determined the dissociation constant for this interaction by performing RNA EMSA with varying amounts of PIP4K2A. By plotting the unbound to bound fraction of the probe, we were able to estimate the dissociation constant for the PIP4K2A and 3′UTR of Rad51 to be around 750 nM (Figure 1B; Supplementary Figure S1A). In order to identify the minimal region of Rad51 3′UTR that is sufficient for interaction with PIP4K2A, we fragmented the RNA into two fragments of 35 and 22 bases. Using these fragments as competitors in RNA EMSA reaction, we determined that the 5′proximal 35 base sequence was sufficient for binding to PIP4K2A (Supplementary Figure S1B). This sequence is predicted to have a stem loop structure (Figure 1C), and we believe that this structure may be important because of its ability to interact with PIP4K2A. We then labeled this fragment (Rad51-short) and performed EMSA experiments to assess the direct interaction of the fragment with PIP4K2A. Incubation of the purified PIP4K2A with the labeled Rad51-short RNA resulted in the formation of a specific shifted band (Figure 1D). The shifted band could be competed out in the presence of unlabeled Rad51-short RNA but not by a nonspecific control RNA. We determined the dissociation constant for Rad51-short RNA and PIP4K2A to be about 500 nM (Figure 1E; Supplementary Figure S1C). We also performed RNA EMSA experiments using an increasing concentration of unlabeled RNA as a competitor and found that the shifted band intensity decreased as we increased the amount of unlabeled self-competitor (Supplementary Figure S2A). PIP4K2A is a kinase that phosphorylates the PI5P at 4^th^ position to generate PI4,5P_2_. We assessed whether the RNA binding activity of PIP4K2A was linked to its function as the phosphorylating enzyme of Phosphatidyl inositol 5-phosphate. We generated and expressed a mutant PIP4K2A (G131L, Y138 F) that was previously shown to lack kinase activity (Bultsma et al., 2010) and assessed its RNA-binding ability. We observed that the RNA-binding ability of mutant PIP4K2A was very similar to that of wild-type PIP4K2A (K_d_ ∼850 nM) with no appreciable change (Figures 1F,G; Supplementary Figure S1D). These results show that the kinase activity of PIP4K2A was not necessary for the RNA binding activity.

### Import of Host PIP4K 2A Is Also Observed in *Plasmodium berghei* and *Toxoplasma Gondii*

**FIGURE 2:**
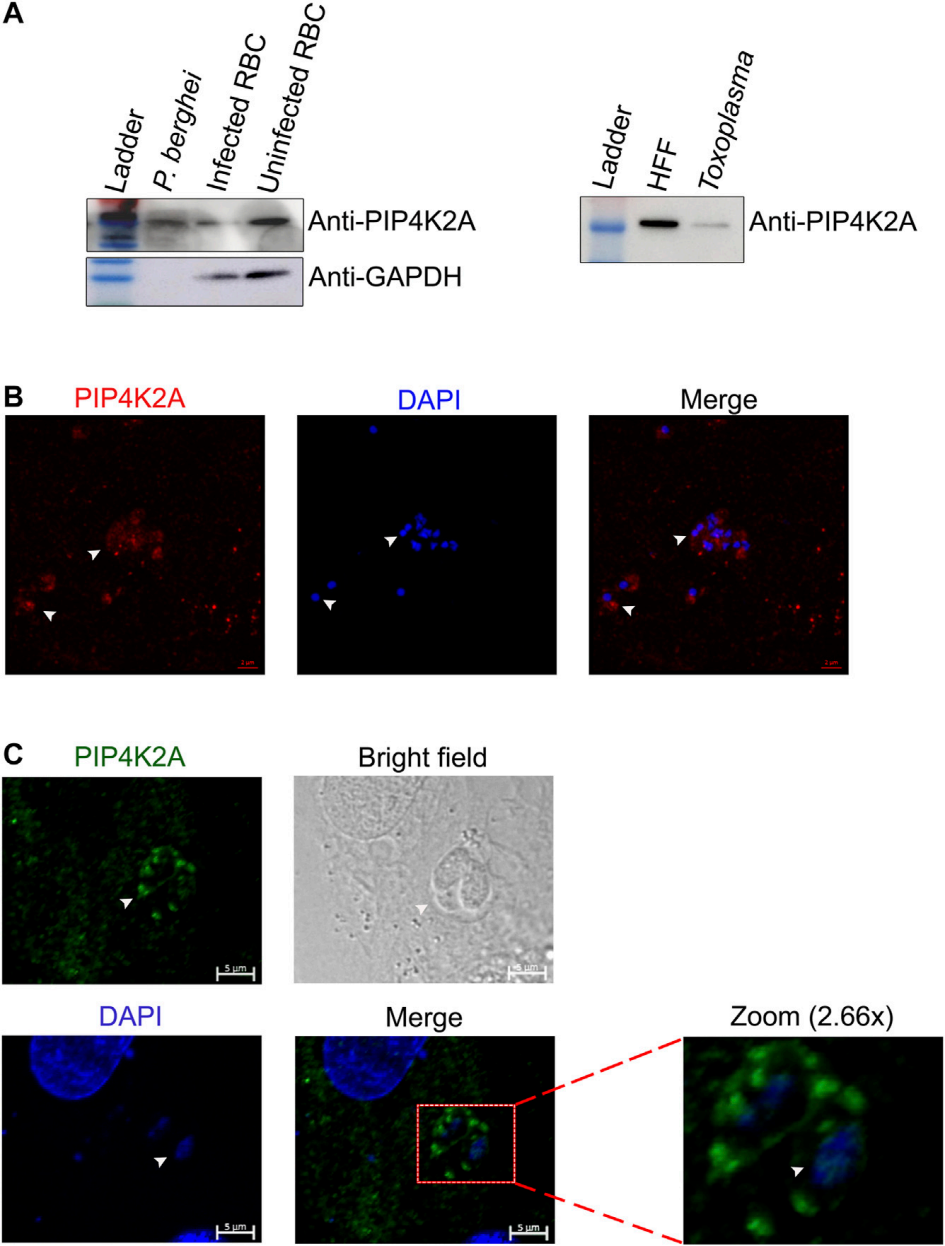
The import of PIP4K2A from a host cell is conserved in other apicomplexa family members. **(A)**. RBC lysate and isolated purified *P. berghei* lysate were resolved on 10% SDS PAGE and probed with anti-PIP4K2A (left top panel), and anti-GAPDH (left lower panel). Human foreskin fibroblast cell (HFF) lysate and purified toxoplasma parasite lysate were resolved on 10% SDS PAGE and probed with anti- PIP4K2A (right panel) (*n* = 3). **(B)** Immunostaining of purified *P. berghei* using anti-PIP4K2A {ab154585 (in red)} and DAPI (in blue). The parasite is indicated by arrows. **(C)** Immunostaining of HFFs infected with toxoplasma using anti-PIP4K2A {(sc100406 (in green)} and DAPI (in blue) (*n* = 3). The arrows indicate the parasite.

We assessed whether importing PIP4K is only restricted to *P. falciparum* or if it is a common feature in other apicomplexans. We assessed whether PIP4K2A is imported from the host cells in *P. berghei*, the mouse malarial parasite, or *T. gondii*, a human parasite. The genomes of both these parasites like *P. falciparum* do not encode the gene for PIP4K2A. We assessed the importation of PIP4K2A from the host cells in these parasites after purifying the parasites. Western blot analysis of the total lysates from both of these parasites showed a specific signal for PIP4K2A in *P. berghei* and *T. gondii* lysate (Figure 2A). The blot was probed with GAPDH (SC-32233) as a control antibody, and a specific signal was detected only in the mouse RBC lysate and not in the *P. berghei* lysate, suggesting that the PIP4K2A signal is not due to contaminating RBC proteins but due to a specific import. The anti-GAPDH also detected GAPDH bands in *T. gondii* lysate, thus we were unable to confirm the purity of the extract. Further, importing PIP4K2A in *P. berghei* and *T. gondii* was assessed by immunostaining. A specific staining for PIP4K2A was observed in the saponin-purified *P. berghei* (Figure 2B) parasite as well in the region of the *T. gondii* parasite just attached to the host human foreskin fibroblast cells (Figure 2C). These results suggest that the importation of PIP4K2A from host cells is not only specific to *P. falciparum* but also observed in other apicomplexans.

### RNA Binding Activity of PIP4K Is Conserved

**FIGURE 3:**
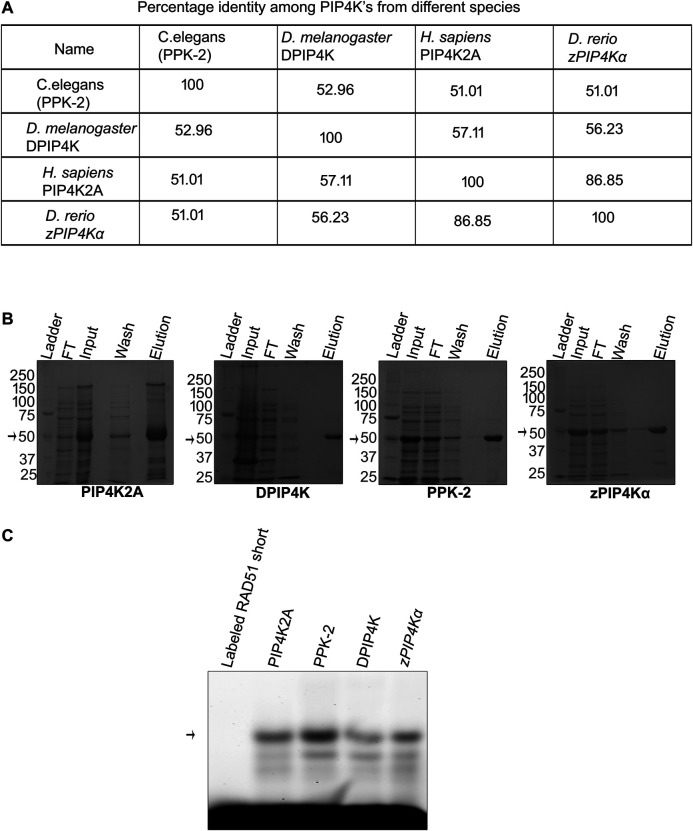
RNA binding activity of PIP4K is conserved among the species. **(A)**. Using ClustalW software, multiple sequence alignment was done, and percentage identity was calculated for different PIP4K’s and is presented in a tabular form. **(B)** Recombinant proteins were induced by 0.5 mM IPTG and purified using nickel chelating resin. Purity was checked on 10% SDS page. **(C)** Recombinant PIP4K2A from human, *D. melanogaster*, *C. elegans*, and *D. rerio* was incubated with labeled RAD51-short for 1 h at 16°C. RNA binding activity was checked by resolving the complex and only labeled RNA on 1% agarose at 4°C (*n* = 3).

Metazoan genome codes for three different isoforms of PIP4K (PIP4K2A, PIP4K2B, and PIP4K2C), while the lower eukaryotes like *Drosophila* code for a single enzyme for this function. We have seen that PIP4K2A has the RNA binding activity, and we wanted to assess if this activity is conserved in PIP4K from other species like *D. melanogaster*, *C. elegans*, and *D. rerio* that have only one isoform of the gene and have between 50 and 80% identity with hPIP4K2A (Figure 3A; Supplementary Figure S3). We expressed and purified PIP4K from these species (Figure 3B), and assessed their ability to bind to Rad51 3′UTR RNA by RNA EMSA. PIP4K from all the species interacted with the *Plasmodium* transcript and was able to form a specific gel-shifted complex (Figure 3C). These results suggest that the RNA binding activity of PIP4K is evolutionarily conserved and may play an important role in gene regulation in these species.

### eIF4EBP1 Interacts With PIP4K2A

**FIGURE 4:**
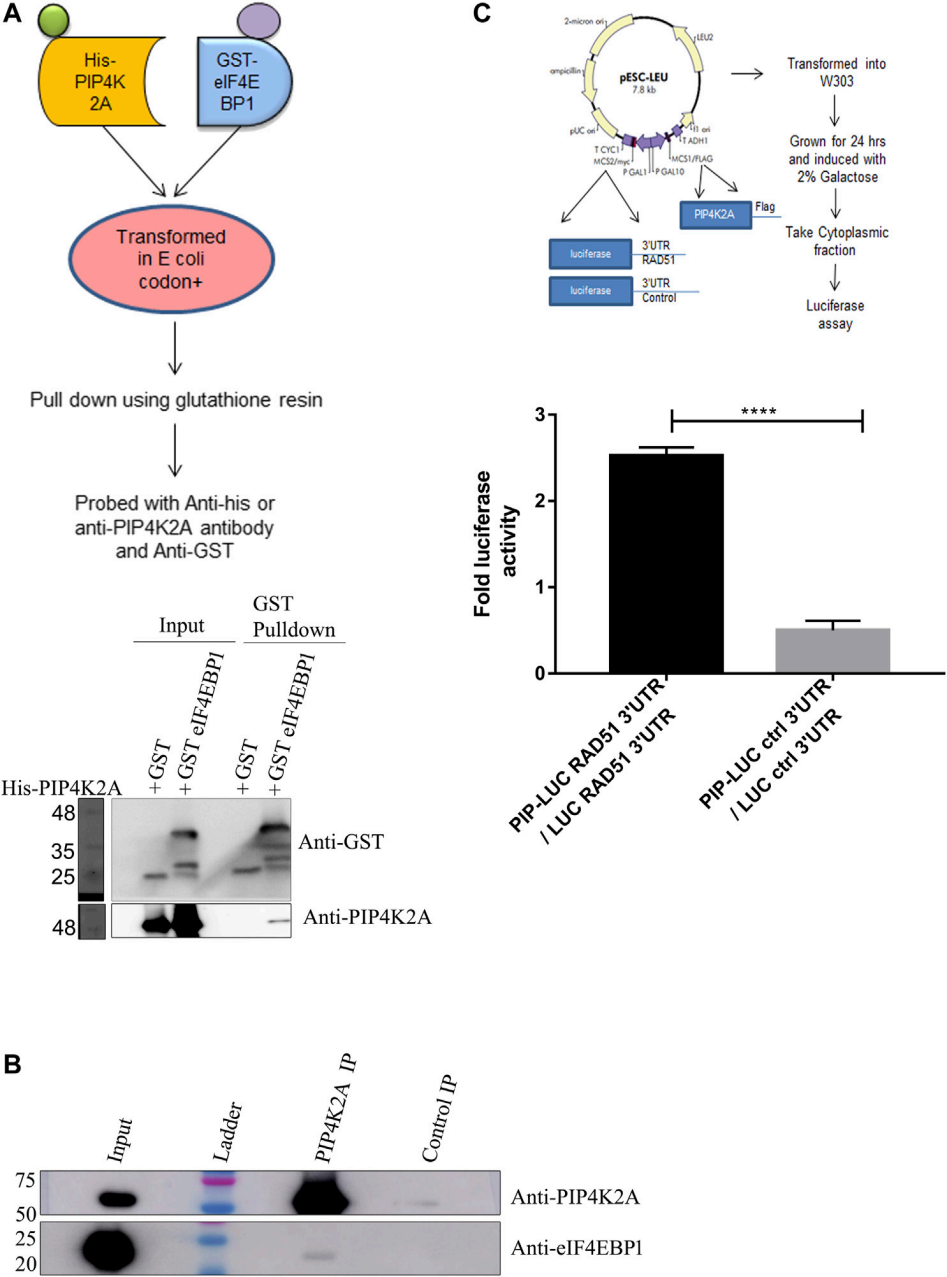
PIP4K2A specifically interacts with eIF4EBP1 and regulates translation. **(A)**. His-PIP4K2A was co-transformed with eIF4EBP1-GST or only GST. Cells were then lysed, and pull-down was performed using glutathione resin. The eluates were then analyzed using SDS-PAGE and Western blotting with antibodies against GST or PIP4K2A (*n* = 3). **(B)**. HEK293 cell lysates were immunoprecipitated with anti-PIP4K2A antibody or control IgG, and the immunoprecipitates were analyzed by Western blotting (*n* = 3). The blots were probed with anti-PIP4K2A (upper panel) and eIF4EBP1 (lower panel). **(C)**. PIP4K2A along with firefly luciferase containing either RAD51 3′UTR or control 3′UTR in pESCLeu vector was transformed into a W303a yeast strain. The transformed yeast cells were grown in Leu selection media and induced with galactose. The cells were then lysed, and the luciferase activity was assessed using Steady-Glo Luciferase assay. Luciferase activity was normalized for the protein content, and the mean (±SEM) fold change in luciferase activity was plotted for luciferase-containing control UTR or Rad51–3′UTR (*n* = 3; unpaired *t*-test, one-tailed, *p* < 0.0001).

Since the RNA binding activity of PIP4K seems to be conserved in species, we believe that PIP4K2A may have additional function apart from its kinase function. To get an idea about its additional role in cells, we tried to identify the proteins that it interacts with. Since PIP4K2A RNA binding activity was discovered in transcripts that are translationally regulated, we analyzed whether PIP4K2A can interact with specific translation factors. We co-expressed various proteins involved in translation as GST fusion proteins along with His-tagged PIP4K2A. We assessed the interaction by using GST beads to isolate the translation factors and assessed whether PIP4K2A was also associated with the factor by the Western blot analysis. We found that among the seven factors tested (Supplementary Figure S4), only recombinant eIF4EBP1 was able to interact specifically with PIP4K2A (Figure 4A). We further validated the interaction of PIP4K2A with eIF4EBP1 by Co-IP using HEK293ET lysates. We observed that a specific band corresponding to eIF4EBP could be detected in PIP4K2A immunoprecipitate (Figure 4B), suggesting specific interaction between PIP4K2A and eIF4EBP1. In order to further delineate the role of PIP4K2A in regulating translation, we performed a luciferase reporter assay in *S. cerevisiae*. We chose the yeast system because yeast does not have an endogenous PIP4K2A gene to confound the assay. We cloned the Rad51 3′UTR downstream to the luciferase open reading frame in the yeast expression vector. PIP4K2A was also cloned in the yeast expression vector, and we performed the luciferase assay to assess the role of PIP4K2A in translation regulation. We found that the expression of PIP4K2A leads to the activation of luciferase activity in the presence of Rad51-UTR compared to the control UTR, suggesting that association of PIP4K2A to the RNA may result in the stimulation of translation (Figure 4C).

## Discussion

It is believed that the main role of PIP4K2A is in regulating the levels of PI5P in mammalian cells. PIP4K2A is predominantly a cytoplasmic protein; however, its substrate is membrane bound. Phosphoinositides and their kinases also play an important role in the growth of *P. falciparum* (Tawk et al., 2010; Ebrahimzadeh et al., 2018; Wengelnik et al., 2018; Joseph et al., 2019). Parasites export several proteins to the erythrocytes that require the interaction of these proteins to lipid-associated phosphatidyl inositol 3 phosphate in the parasite endoplasmic reticulum. Proteins regulating these have been identified as a specific targets for drugs against malaria (Vaid et al., 2010; Mbengue et al., 2015). We have shown that one member of this pathway, that is, PIP4K2A, is imported into the parasite from the host where it associates with a specific parasite RNA. We show that the interaction of PIP4K2A with the RNA is strong with a K_d_ of about 750 nM, suggesting that PIP4K2A and RNA interaction could occur in the physiological range of protein concentration. We further identified a shorter fragment of RNA that was sufficient for interaction with PIP4K2A. This short fragment is predicted to form a stable stem loop secondary structure with multiple UUGU motifs. This motif has been previously shown to be important for interaction with the Puf family of RNA-binding proteins (Cui, 2002). This suggests a possibility that PIP4K2A could co-regulate a subpopulation of transcripts that are regulated by Puf proteins. In the case of PIP4K2A, the relationship between the RNA binding activity and the kinase activity seems to be independent; however, it is possible that RNA binding may regulate its interaction with other proteins and may affect the localization of the kinase. We believe that some of the kinase-independent functions of PIP4K (Kamalesh et al., 2017; Sharma et al., 2019) could be attributed to its RNA binding activity; thus, the RNA binding activity of PIP4K2A may be an important function of PIP4K2A apart from its role in phosphoinositide’s metabolism.

Importation of PIP4K2A from the host cells is conserved among the three protozoan parasites whose genomes lack the PIP4K gene, namely, *P. falciparum*, *P. berghei*, and *T. gondii.* This suggests that the host PIP4K2A may be performing an important function in the parasite that increases their fitness levels. We had previously reported that the depletion of PIP4K2A from the host cells results in reduced parasitemia in the case of *P. falciparum* (Vindu et al., 2018). The RNA binding activity of PIP4K seems to be conserved as PIP4K from lower eukaryotes also showed this activity. This suggests that the RNA binding activity of PIP4K could be important for the posttranscriptional gene regulation. Although we show that PIP4K2A interacts with few specific parasite RNA, it is possible that it may bind to other targets in both the host and the parasite.

**FIGURE 5:**
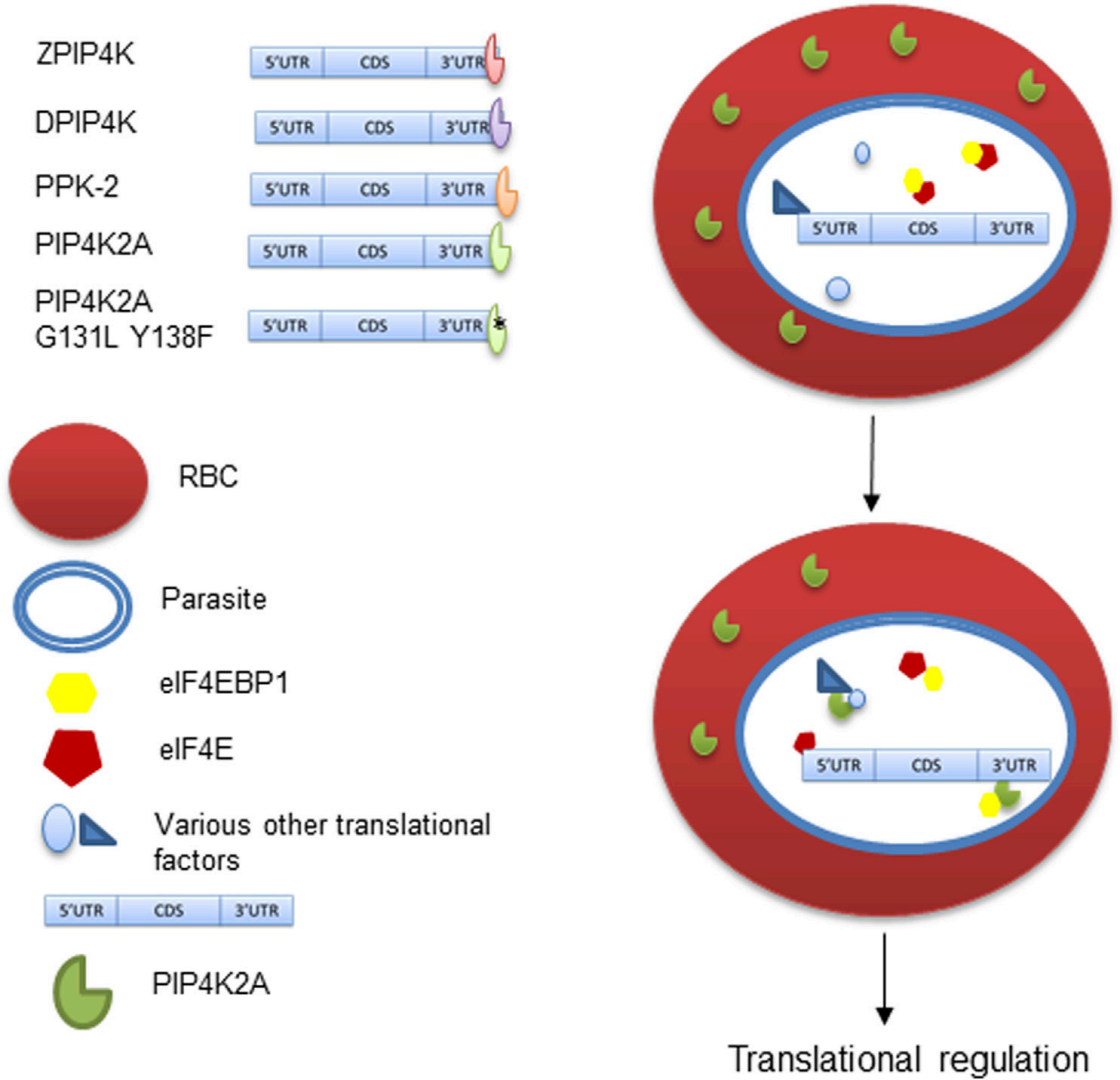
RNA binding activity of PIP4K and its role in translation regulation. PIP4K from different species have RNA binding activity and bind to 3′UTR sequence. The RNA binding activity is independent of its kinase activity in PIP4K2A. PIP4K2A can specifically interact and sequester eIF4EBP1, which is a known translational repressor. This in turn makes eIF4E available for mRNA cap binding and translation initiation.

PIP4K2A physically interacts with eIF4EBP1 protein suggesting that recruitment of translation factors to the mRNA could be regulated by PIP4K2A. eIF4EBP1 binds with the cap-binding factor eIF4E, thereby inhibiting translation. PIP4K2A could act as a local sponge for eIF4EBP1, thereby promoting translation of the specific mRNA with which PIP4K2A is associated (Figure 5). This is supported by the luciferase assay that shows that PIP4K2A can specifically enhance the translation of transcripts containing its binding target. Both PIP4K2A and eIF4EBP1 have been shown to be involved in tumor progression (Cha et al., 2015), suggesting a common link between the two. We believe that apart from its role as phosphatidylinositol kinase, PIP4K2A may have a role in regulating posttranscriptional gene expression either by altering the translation of the RNA or its stability.

## Data Availability

The original contributions presented in the study are included in the article/Supplementary Material; further inquiries can be directed to the corresponding author.
