# Supplementary material for: Conserved RNA Binding Activity of Phosphatidyl Inositol 5-Phosphate 4-Kinase (PIP4K2A)
Source: Front Mol Biosci. 2021 May 28;8:631281. doi: 10.3389/fmolb.2021.631281 (PMC8194828; doi:10.3389/fmolb.2021.631281)
Supplement: Supplementary file 1 [file datasheet1.pdf]

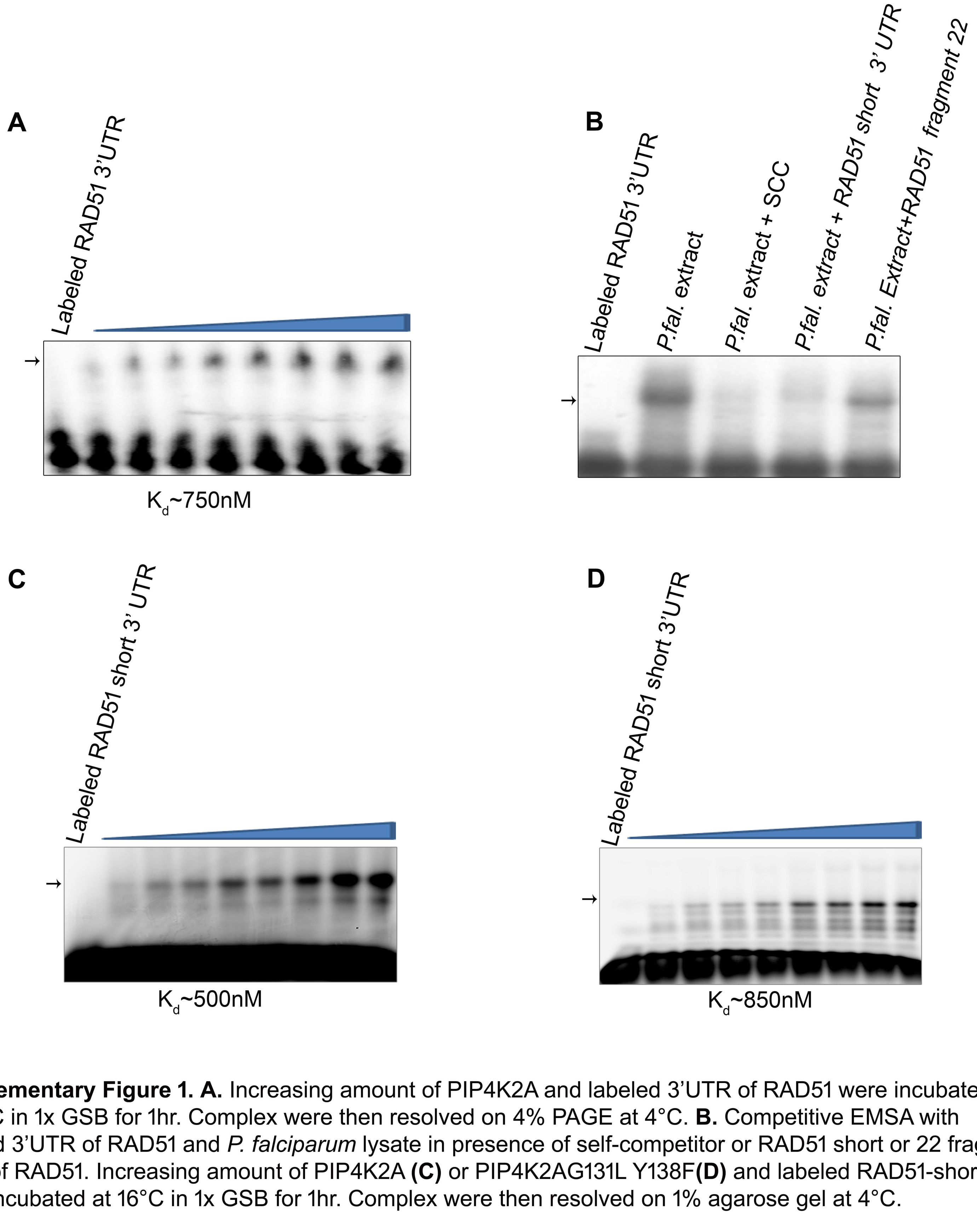

Supplementary Figure 1. A. Increasing amount of PIP4K2A and labeled 3'UTR of RAD51 were incubated at 16°C in 1x GSB for 1hr. Complex were then resolved on 4% PAGE at 4°C. **B.** Competitive EMSA with labeled 3'UTR of RAD51 and *P. falciparum* lysate in presence of self-competitor or RAD51 short or 22 fragment of RAD51. Increasing amount of PIP4K2A **(C)** or PIP4K2AG131L Y138F**(D)** and labeled RAD51-short were incubated at 16°C in 1x GSB for 1hr. Complex were then resolved on 1% agarose gel at 4°C.

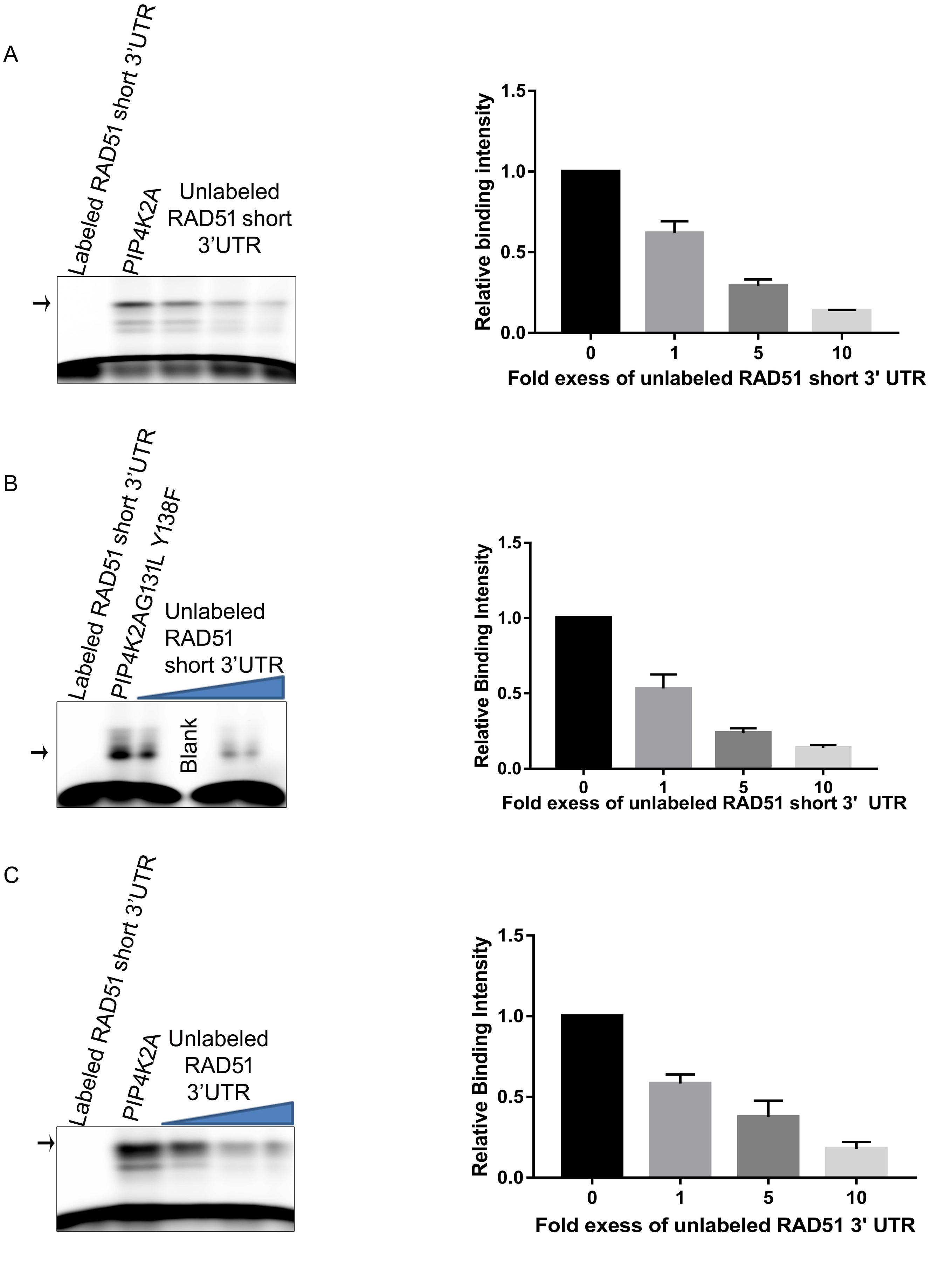

Supplementary Figure 2. A/C) Increasing amount of unlabeled RAD51 3'UTR short/ 3'UTR of RAD51 along with labeled 3'UTR of RAD51 short and PIP4K2A were incubated at 16°C in 1x GSB for 1hr. Complex were then resolved on 1% agarose at 4°C. The shifted bands were quantified using image J software and the mean (± SEM) relative band intensity was plotted (right panel; n=3). **B)** Increasing amount of unlabeled RAD51 3'UTR short along with labeled 3'UTR of RAD51 short and PIP4K2AG131L Y138F were incubated at 16°C in 1x GSB for 1hr. Complex were then resolved on 1% agarose at 4°C. The shifted bands were quantified using image J software and the mean (± SEM) relative band intensity was plotted (right panel; n=3).

```
       C. elegans  ------MSTKKKTKVLSKKKGKILVPKWKLFRAKEPVLSVFMWGINHTVDQLLHVPPPGL 54
D. melanogaster  ME--KKISSSSQPRILKKKHFRVKHQKVKLFRANEPILSVFMWGINHTINELSHVNIPVM 58
       H. sapiens  MATPGNLGSSVLASKTKTKKKHFVAQKVKLFRASDPLLSVLMWGVNHSINELSHVQIPVM 60
          D. rerio  MASA-SNTVSSFASKTKTKKKHFVAQKVKLFRASDPLLSVLMWGINHSINELSHVQIPIM 59
                            .      ..*: :.   * *****.:*:***:***:**::::* **  * :

       C. elegans  LMPDDFKAYSKVKIDNHNFNKDIMPSHYKVKEYCPNVFRNLREQFGVDNFEYLRSLTSYE 114
D. melanogaster  LLPDDFRAYSKIKVDNHLFNKENMPSHFKVKEYCPLVFRNLRERFGVDDVDYRESLTRSQ 118
       H. sapiens  LMPDDFKAYSKIKVDNHLFNKENMPSHFKFKEYCPMVFRNLRERFGIDDQDFQNSLTRSA 120
          D. rerio  LMPDDFKAYSKIKVDNHLFNKENMPSHFKFKEYCPLVFRNLRERFVIDDQDFQNSLTRSA 119
                   *:****:****:*:*** ***: ****:*.***** *******:* :*: :: .***

       C. elegans  PEPDLLDGSAKDSTPRFFISYDKKFVIKSMDSEAVAELHSVLRNYHQYVVEKQGKTLLPQ 174
D. melanogaster  PIQI---DSSGKSGAQFYQSYDKFFIIKSLTSEEIERMHAFLKQYHPYVVERHGKTLLPQ 175
       H. sapiens  PLPN---DSQARSGARFHTSYDKRYIIKTITSEDVAEMHNILKKYHQYIVECHGITLLPQ 177
          D. rerio  PLVS---EAQGRSGARFHTSYDKRYVIKTISSEDVAEMHNILKKYHQYIVECHGSTLLPQ 176
                   *       :   *  :*. **** ::**:: ** :  .:*  .*::** *:** :* *****

       C. elegans  YLGLYRLTIEGSETYLIVMRNVFGRKYGVHTKFDLKGSTVSRAASDKEKAKDLPTLKDND 234
D. melanogaster  YLGMYRITVESVQYYFVVMRNVFSSHLTIHKKFDLKGSTVDREASEKELEKNLPTFKDND 235
       H. sapiens  FLGMYRLNVDGVEIYVIVTRNVFSHRLSVYRKYDLKGSTVAREASDKEKAKELPTLKDND 237
          D. rerio  FLGMYRLTVDGDETYMIVTRNVFSHRLSVYKKYDLKGSTVAREASDKEKAKELPTYKDND 236
                   :**:**:.::. : *.:* ****. :  :: *:******* * **:**  *:*** ****

       C. elegans  FLEQNWKLNLPPEAGKLLIEMLTSDTEWLTRMHLMDYSLLVGIHDCERAAQEAANRPV-- 292
D. melanogaster  FIKQKVKLDIGKEAKDKLMDTLSNDVDLLTKLHIMDYSLLVGVHDCVRAEEEALQGDNIL 295
       H. sapiens  FINEGQKIYIDDNNKKVFLEKLKKDVEFLAQLKLMDYSLLVGIHDVERAEQEEVECEE-- 295
          D. rerio  FINDGQKIYIDDESKKIFLNKLQKDVEFLALLKLMDYSLLVGIHDVERAEQEEVESED-- 294
                   *:::  *: :  :  . ::: * .*.: *: :::********:**  ** :*  :

       C. elegans  ---------EQNSEESGDELAPTPPDSPIPSTGGAFPGVSGGPDLDDEFYAIASPADFEK 343
D. melanogaster  TVGRSENSESEECDSGERWTYNTPPDSPRGAQYK-------EVVYEVDIYDIPSI--EEK 346
       H. sapiens  ------NDGEEEGESDGTHPVGTPPDSPGNTLNSSPPLAPGEFDPNIDVYGIKCHENSPR 349
          D. rerio  ------NEGEEEGESDGG-IVGTPPDSPSNTLDSTKPLSPGDFDPNIDVYAIKSHDNSPR 347
                            .:: :..      ******  :              : :.* * .     :

       C. elegans  NLIYFIGLVDILTYYGIKKRSATAAKTVKYGSDAENISTVKPEQYAKRLVEFVSRALN   401
D. melanogaster  REIYFIAIIDVLTQYGVKKQAAKAAKTVKYGSNVDGISTCDPEQYAKRFLDFMDKAIE   404
       H. sapiens  KEVYFMAIIDILTHYDAKKKAAHAAKTVKHGAGA-EISTVNPEQYSKRFLDFIGHILT   406
          D. rerio  KEVYFMAVIDILQHYDAKKKAAHAAKTVKHGAGA-EISTVNPEQYSKRFYDFITTILP   404
                   . :**:.::*:*  *. **::* ******:*:..  *** .****:**: :*:   :
```

Supplementary Figure S3: Protein alignment of PIP4K from worm (*C. elegans*), fly (*D. melanogastor*, mamal (*H. sapiens*) and fish (*D. Rerio*), using ClustalW

Translation factor	eIF4EBP1	eIF4E	PAIP1	PAIP 2	YBX1	eIF4G 1920	eIF4A1
Interaction with PIP4K2A	+	–	–	–	–	–	–

Supplementary Figure 4. Table showing translation factors tested for interaction with PIP4K2A
